# An experimental and computational study of graphene oxide functionalized with tris(hydroxymethyl)aminomethane as an electrode material for supercapacitors

**DOI:** 10.1038/s41598-023-44048-z

**Published:** 2023-10-05

**Authors:** Samira Mohammadi, S. Morteza Mousavi-Khoshdel

**Affiliations:** Industrial Electrochemical Research Laboratory, Department of Chemistry, Iran University of Science and Technology, Tehran, Iran

**Keywords:** Chemistry, Energy science and technology, Materials science, Nanoscience and technology

## Abstract

In this research, graphene oxide (GO) functionalized with tris(hydroxymethyl)aminomethane (T) was synthesized with a simple one-pot method, and applied as an electrode material for supercapacitors. Electrochemical measurements on the synthesized tris(hydroxymethyl)aminomethane-functionalized graphene oxide (GO@T) indicated a specific capacitance of 549.8 F g^− 1^ at a specific current of 2.5 A g^− 1^ and a specific capacitance of 358 F g^−1^ at a specific current of 7 A g^− 1^ in the potential range of − 0.5–0.5 V versus Ag/AgCl. It also showed a high cyclic stability. According to the results, 80 and 68% of the initial capacitance was retained after 5500 and 9300 cycles, respectively. Density functional theory calculations were used to investigate the quantum capacitance, free energy change during functionalization reaction, and the layer distance of GO and GO@T.

## Introduction

Electric energy storage devices are of high importance for mobile electrical devices, energy consumption management, and coupling with unsustainable renewable energies^1–3^. Nowadays, the development of electrical energy storage devices is vital for the portable energy consumer and its coupling with energy production units. The performance of the energy storage device has an important effect on the performance of the systems that use electrochemical storage devices^4,5^. Electrochemical supercapacitors have drawn much consideration in recent years due to their durable cycle life, fast charging and discharging, wide operating temperature range, high power density, reversibility, and low environmental impact which make them suitable for various applications such as uninterruptible power sources (UPS) and coupling with batteries in electric vehicles^3,6,7^. They can be partitioned into two basic types according to their charge storage mechanism; (i) Electrical double layer capacitance (EDLC), and (ii) Faraday capacitance (pseudo capacitance). EDLC relates to charge separation at the electrode/electrolyte interface which makes long-term stability and fast charge–discharge cycles which is mainly based on carbon materials (due to their high surface area). On the other hand, pseudo capacitance arises from fast faradic reactions in the electrode mainly consisted of transition metal oxides and conducting polymers which leads to high energy densities. The combination of EDLC and pseudo capacitance in materials have significantly increased the capacitance and efficiency of supercapacitors^8,9^.

Electrode materials should provide proper chemical stability, corrosion resistance, thermal stability, large specific surface area, high electrical conductivity, environmentally friendliness, and economic efficiency^10,11^. Graphene is the most attractive material for supercapacitor electrode, due to its high specific surface area, excellent stability, high conductivity, high power density, and electrochemical properties^12,13^. Among the graphene derivatives, graphene oxide (GO) has recently received more attention for supercapacitor applications due to its easy preparation and better dispersing compared to graphene^14^. For example BinSabt showed that the specific capacitance of β-Ni(OH)_2_/graphene oxide increases compared with β-Ni(OH)_2_^15^.

Van der Waals interactions cause aggregation in graphene and graphene oxide, which reduces its surface area and consequently decreases the double layer capacitance. Thus, there are different methods to overcome the re-stacking of graphene, including doping, creating defects, and functionalizing with organic and mineral compounds^14,16–20^. Shi has constructed organic molecule-based electrodes by combining redox-active organic molecules with conductive carbon-based materials such as graphene^21^. Czepa has reported an unprecedented approach for GO modification with thioamide-based polymers featuring numerous heteroatoms (S, N, O), which is useful for achieving superior electrochemical performance in symmetric supercapacitors^22^. Dashti Najafi functionalized graphene oxides with cytidine triphosphate and arabinosylcytosine and used them as supercapacitor^23^.

Tris(hydroxymethyl)aminomethane, known as tris, is a small organic molecule that comprises one amino group and three hydroxyl groups. It is a non-toxic material that is used for biochemistry and medicine^24^. Tris has been recently used for vanadium redox flow battery electrode material It has wide potential window but its electrical conductivity is low^25^.

In this study, tris(hydroxymethyl)aminomethane-functionalized graphene oxide (GO@T) is synthesize through a simple one-pot method as a new electrode material for supercapacitors. Tris, as a non-toxic organic compound, was placed on the surface of graphene oxide through an amide reaction in order to prevent GO accumulation and thus, achieve higher specific surface area for the GO. Therefore, the electrochemical behavior of GO@T was improved compared to GO and tris, so that the assembled GO@T electrode exhibited a high specific capacitance of 549.8 F g^− 1^ at a specific current of 2.5 A g^− 1^. DFT calculation was also used to investigate the functionalized tris, the distances between the GO layers, and the diagrams of free energy change during functionalization reaction. Also, the quantum capacitance of GO@T and GO was calculated. This work presents a low-cost, efficient, and non-toxic material for use in supercapacitors and it can be considered as an important step towards sustainable development.

## Results and discussion

### Material characterization

Characterization of tris-functionalized graphene oxide was carried out using Fourier-transform infrared (FT‐IR) spectroscopy (Shimadzu 8400 s), X-ray diffraction (XRD, TW 1800 diffractometer with Cu _Ka_ radiation (λ = 1.54050 Å)), energy dispersive X-ray analysis (EDX) on a TESCAN electron microscope device (model 4992), field emission scanning electron microscopy (FESEM) (TESCAN-MIRA3), and thermal gravimetric analysis (Bahr Company STA 504).

### Characterization of the combinations

**Figure 1:**
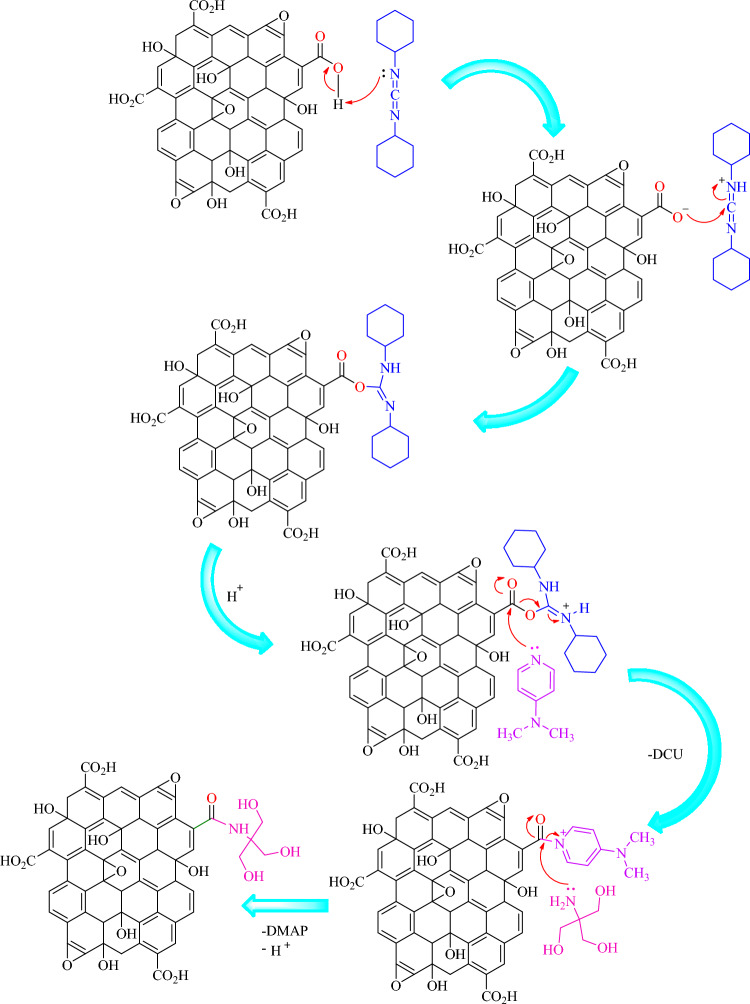
The amide reaction mechanism of GO@T.

In this study, a simple one-pot method was used for the synthesis of tris-functionalized graphene oxide. Dicyclohexylcarbodiimide (DCC) reacts with graphene oxide, leading to the elimination of H^+^ from acidic group. In the next step, surface oxygen groups are activated for nucleophilic attack. The negative charge attacks the carbon of DCC and the double bond is opened. The nitrogen atom of imine is activated by taking a proton from the environment and gets a positive charge. At this stage, 4-dimethylaminopyridine (DMAP) attacks the carbon of carbonyl as a nucleophile and the electrons of the double bond get a shift to oxygen atom and these electrons in return removes the dicyclohexylurea (DCU) group. Tris also attacks the carbon of carbonyl as a nucleophile and the electrons of the double bond get a shift to oxygen atom and this electron in return removes the DMAP group, leading to the neutralization of the tris. Figure 1 demonstrates the amide reaction mechanism^26–28^.

**Figure 2:**
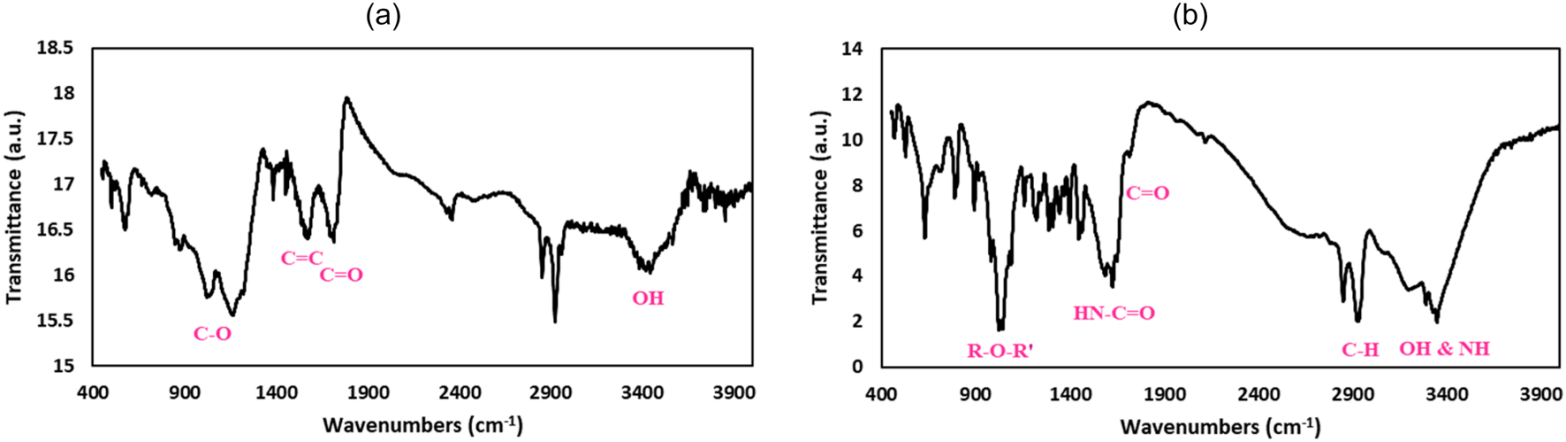
FTIR spectra of (**a**) GO and (**b**) GO@T.

FT-IR spectrum of the synthesized GO is shown in Fig. 2a. The peak at 1717 cm^− 1^ is related to the stretching vibration of the C=O carbonyl group. Also, the presence of the peak at 1576 cm^− 1^ is associated with the C=C double bond. The peaks observed at 1165 and 1024 cm^− 1^ are related to the C–O bond of epoxide and ether, respectively. The presence of a broad peak in the range of 3100–3400 cm^− 1^ is also related to the OH groups^29–31^.

As shown in the FTIR spectra of GO@T in Fig. 2b, the absorption band in the range of 3000–3400 cm^− 1^ is related to O–H and N–H groups, also the adsorption band at 2932 cm^− 1^ is corresponding to the C–H aliphatic bonds in tris. The adsorption band at 1628 cm^− 1^ belongs to the amide (HN–C=O) groups, indicating that GO was successfully functionalized by tris. In addition, the adsorption band at 1024 cm^− 1^ belongs to the epoxy group^32^.

**Figure 3:**
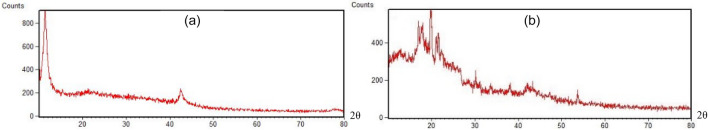
XRD patterns of (**a**) GO and (**b**) GO@T.

XRD patterns of pure graphene oxide are shown in Fig. 3a. The peaks at 2θ of 11.38°, 15.37° and 42.45° confirm successful synthesis of GO^30,33^.

Figure 3b shows XRD patterns of tris-modified graphene oxide. The broad peak in the 2θ range of 20–30° is related to GO. The sharp peaks at 16.97°, 17.77° and 19.89° are related to GO, while the additional peaks observed in the 2θ range of 21.11°, 21.53°, 30.13°, 38.12°, 43.31°, and 53.71° are characteristic of the tris structure which proves successful functionalization of GO (JCPDS No: 00-050-2098).

**Figure 4:**
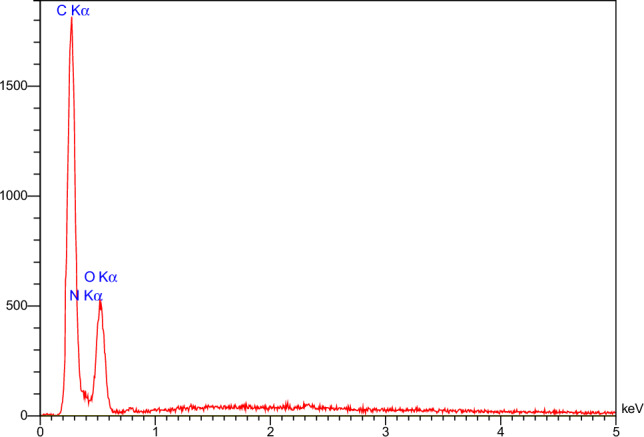
EDX spectra of GO@T.

EDX analysis was performed to further confirm the structure of the prepared GO@T. As shown in Fig. 4, the structure of the GO@T mainly consists of C (47.35%), O (31.46%), and N (21.19%) elements.

**Figure 5:**
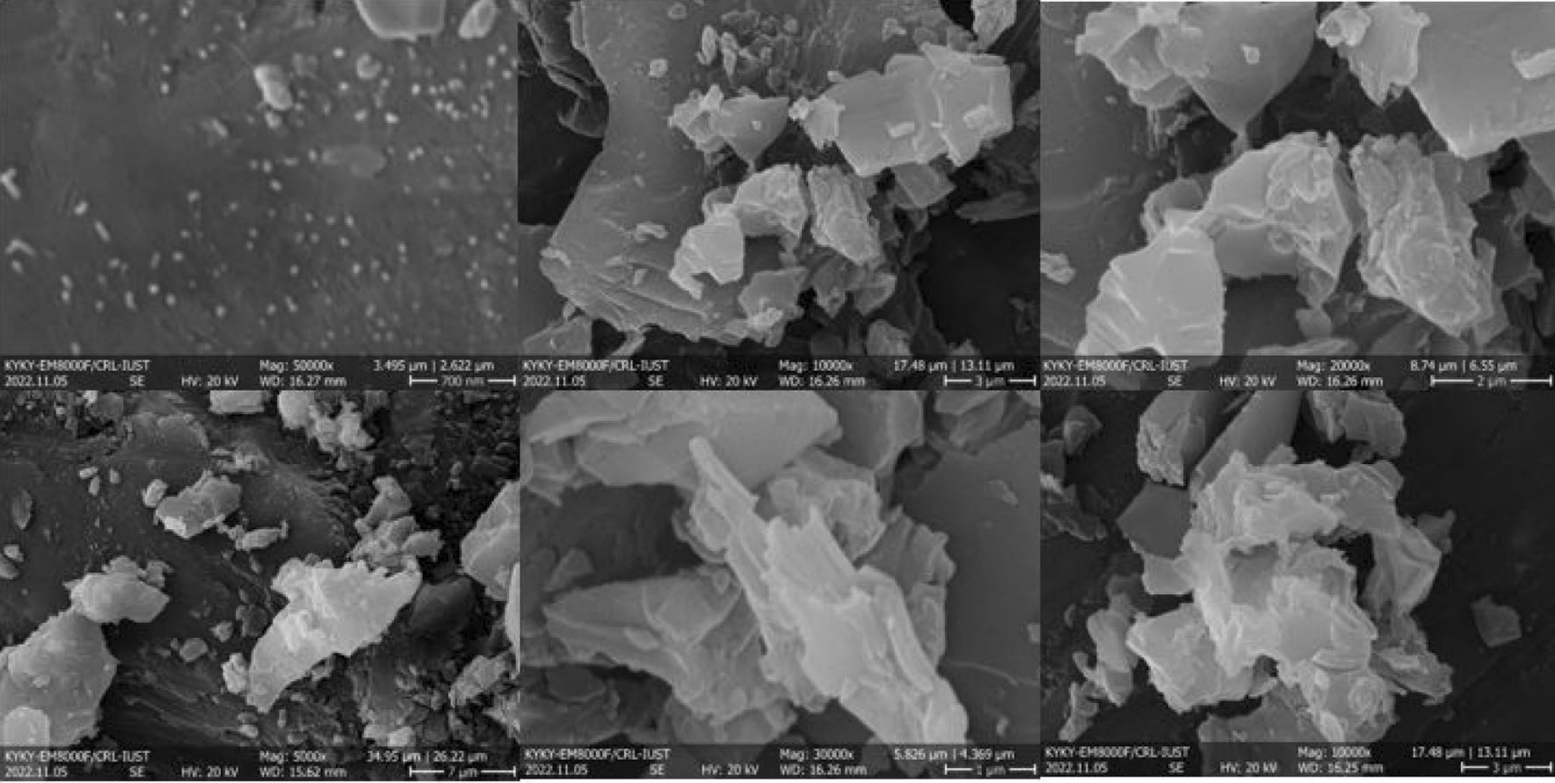
FESEM images of GO@T.

FESEM images of GO@T are illustrated in Fig. 5 to investigate the surface morphology of the GO@T composite. It is evident that the GO sheets have changed from uniform and regular morphology to twisted and irregular one, confirming that the final composite has been successfully synthesized.

### Electrochemical investigation of the supercapacitor

#### Cyclic voltammetry

**Figure 6:**
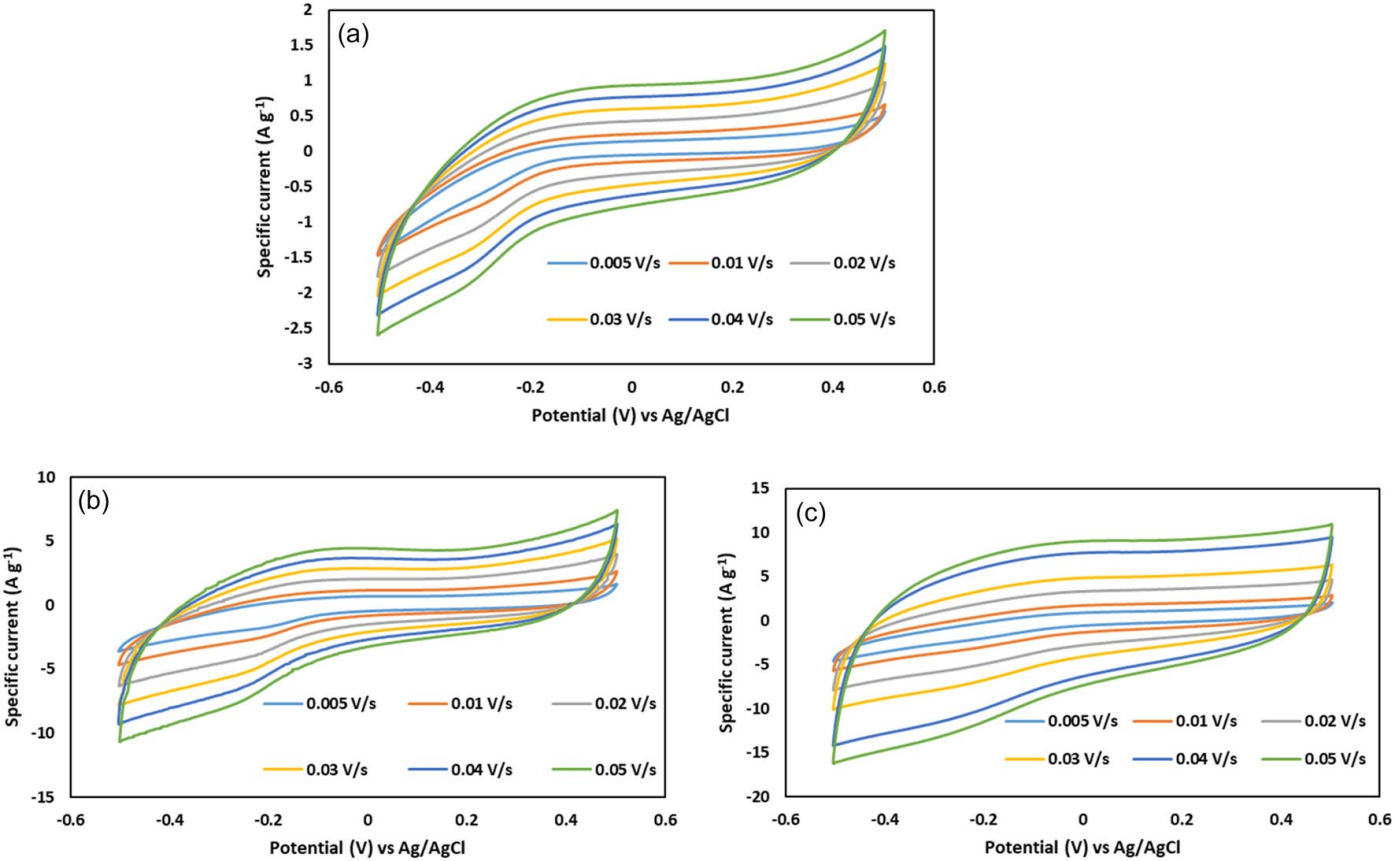
CV curves obtained at different scan rates for (**a**) tris, (**b**) GO and (**c**) GO@T.

The cyclic voltammetry (CV) of the species in different scan rates and − 0.5–0.5 V versus Ag/AgCl potential range is shown in Fig. 6. As shown in Fig. 6a, there is no peak in the potential window (− 0.5–0.5 V vs. Ag/AgCl) for the tris and there isn’t any faradic reaction. The shape of the CV curves for GO and GO@T are quasi-rectangular, indicating that no redox reaction has occurred^34^.

The specific capacitance of electrodes is proportional to the area of CV curve according to Eq. (1):

1 $${C}_{m}=\frac{A}{(m\times v\times \Delta V)}$$

where C_m_ is the specific capacitance (F g^− 1^), A is the integrated area of CV, υ is the scan rate (V s^− 1^), m is the mass of the electroactive materials (g), and ΔV is the sweep potential window (V)^35^.

**Figure 7:**
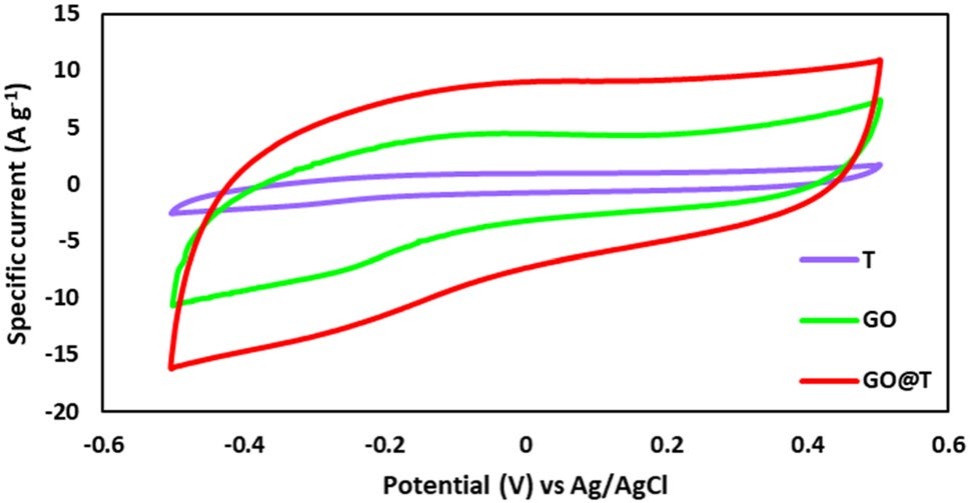
CV curves of T, GO and GO@T at the scan rate of 0.05 V s^− 1^.

The voltammogram of T, GO, and GO@T electrodes with scan rate of 0.05 V s^− 1^ is shown in Fig. 7. As it is clear from the curves, the surface area under CV curve of functionalized GO is much higher than that of other species.

#### Galvanostatic charge/discharge

**Figure 8:**
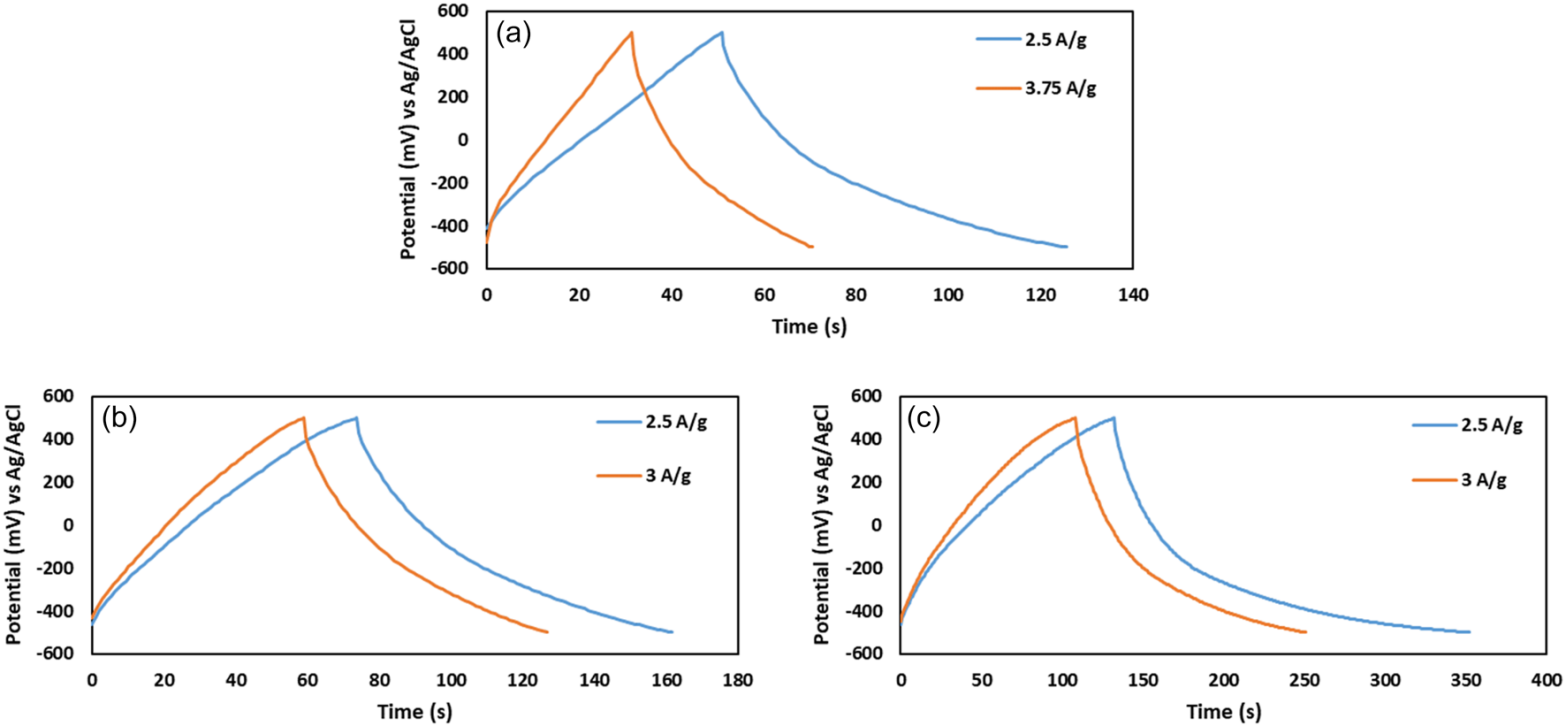
Galvanostatic charge/discharge diagram of (**a**) tris, (**b**) GO and (**c**) GO@T at low specific currents.

The galvanostatic charge/discharge (GCD) curves of samples showed a triangular shape that confirms the EDLC behavior of the electrodes (Fig. 8).

The specific capacitance of the electrode (C_m_) was calculated using Eq. (2):

2 $${C}_{m}=\frac{(i\times \Delta t)}{(m\times \Delta V)}$$

where *i* is the current applied to the electrode for charging or discharging (A), ∆t is the charge or discharge time (s), ∆V is the potential window (V), and m is the total mass of the active materials (g)^36^.

According to Eq. (2), the specific capacitance of T, GO, and GO@T were obtained 186.5, 219.5, and 549.8 F g^− 1^ at a specific current of 2.5 A g^− 1^, respectively. The significant increase in the capacitance of the composite can be due to the better dispersion of tris-functionalized graphene oxide sheets in comparison with GO.

#### Specific capacitance and cyclic stability of GO@T composite

**Figure 9:**
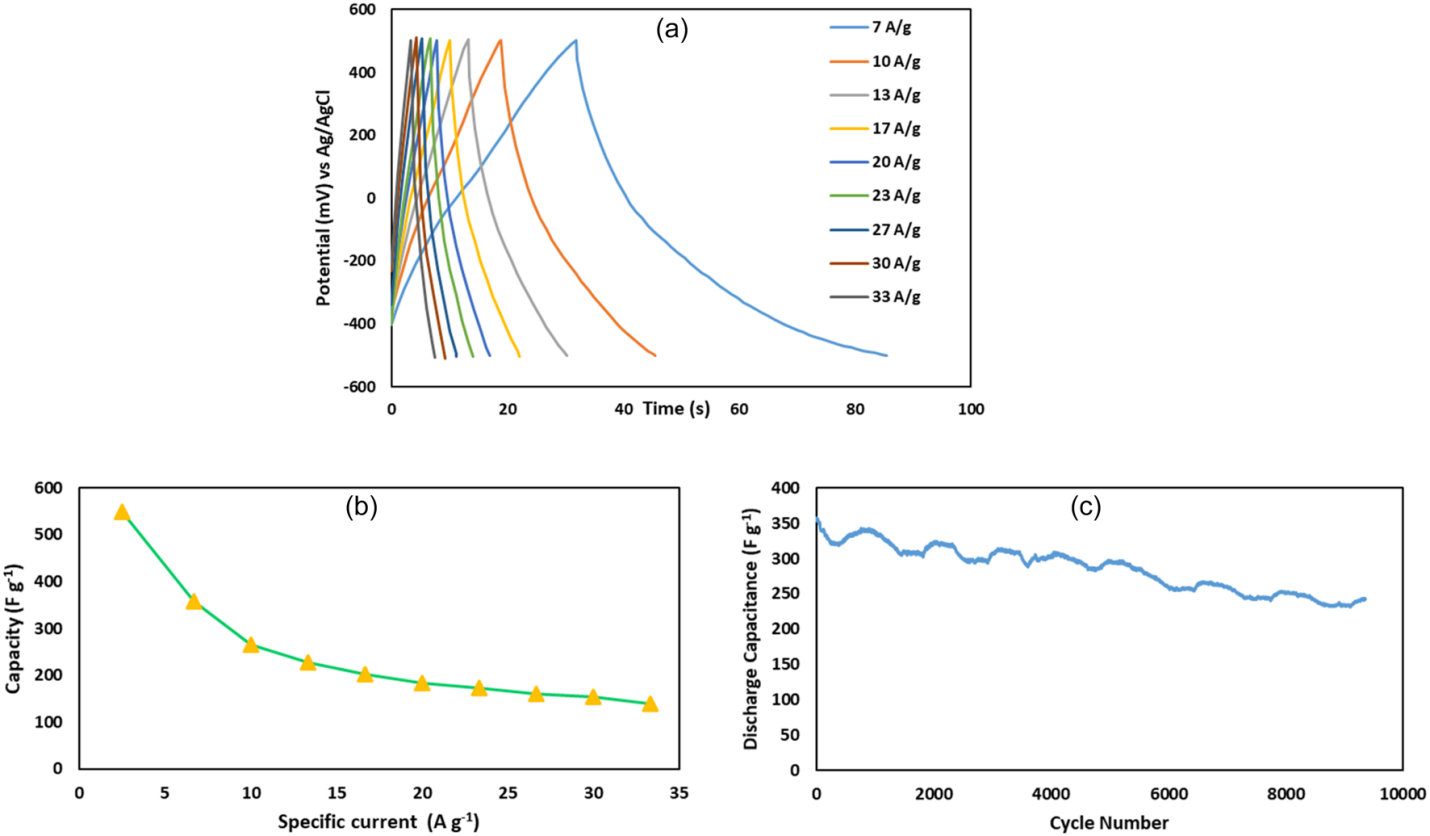
(**a**) Galvanostatic charge/discharge diagram of GO@T at high specific current, (**b**) specific capacity vs. various specific currents, and (c) cyclic stability of GO@T electrode at the specific current of 7 A g^− 1^.

According to the significant improvement in the capacitance of composite electrode, GCD at high specific current (7–33 A g^− 1^) was tested only for GO@T. The resulting GCD curves show a triangular shape and EDLC behavior, as shown in Fig. 9a.

The specific capacitance of GO@T composite at various current densities is shown in Fig. 9b. According to the results, with increasing the specific current from 2.5 to 33 A g^− 1^, the capacity decreased from 549.8 to 140.0 F g^− 1^. This is a common phenomenon which is caused by the insufficient time available for ion diffusion and adsorption inside the small pores within a particle at high specific current. Under these conditions, the diffusion rates of electrolyte ions are limited by electrode structural properties and only the external sites can take part in ion transfer reaction. But at low scan rates, all the active areas, including external and internal surfaces, can be utilized for charge/discharge and electrochemical processes^37,38^. The cyclic stability of GO@T electrode was measured at the constant specific current of 7 A g^− 1^, as shown in Fig. 9c. After 5500 and 9300 cycles, 80 and 68% of the initial capacity was retained.

For the electrochemical supercapacitors, the two most important parameters are energy and power density which are calculated from Eqs. (3) and (4):

3 $$E=\frac{{C}_{m}({\Delta V}^{2})}{7.2}$$

4 $$P=\frac{3600 E}{\Delta t}$$

where E is the energy density (W h kg^− 1^), P is the power density (W kg^− 1^), ΔV is potential range (V), and Δt is the discharge time (s)^39^. The obtained values for energy density and power density were 76.35 W h kg^− 1^ and 1250 W kg^− 1^, respectively.

#### Electrochemical impedance spectroscopy

**Figure 10:**
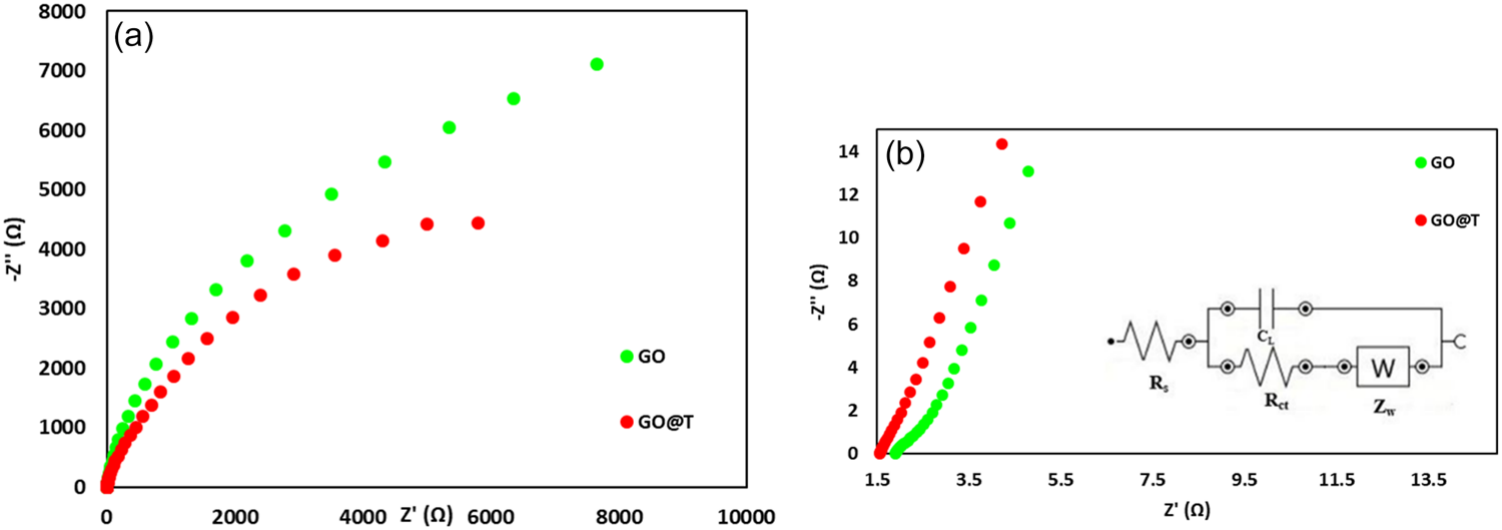
(**a**) Nyquist plots, (**b**) higher magnification of the Nyquist plots at high frequencies and the electrical equivalent circuit.

The Nyquist plots of GO and GO@T electrodes are shown in Fig. 10a, where the imaginary part of the impedance (Z^″^) is drawn versus the real part (Z) in the range between 0.01 Hz and 100 kHz at open circuit potential (− 0.013 V vs. Ag/AgCl). Impedance spectra are included a semicircle in high frequency and a straight line in the low frequency. Figure 10b represents the higher magnification of the Nyquist curve at high frequencies. The electrical equivalent circuit is also shown in Fig. 10b. In this circuit, R_s_, R_ct_, and C_L_ indicate the solution resistance, charge transfer resistance, and the double-layer capacitance, respectively. W is the Warburg element that indicates the electrolyte ion diffusion into the electrode material^40^. Solution resistance for GO and GO@T electrodes was 2.11, 1.66 Ω and those of charge transfer resistance was 2.55, 2.08 μ Ω, respectively.

As can be seen, due to the presence of tris in the composite structure, the charge transfer and solution resistances are reduced in the composite electrode compared to the graphene oxide electrode. This reduction in resistance value increases the electrochemical efficiency of the composite in comparison with the graphene oxide electrode. The linear region of the Nyquist plot depicts the Warburg diffusion element. It can be seen that the composite electrode has a purer capacitive behavior than the GO electrode at low frequencies. It is well known that the larger slope of the linear region of the Nyquist plot is indicative of purer capacitive behavior, shorter diffusion path, and better access to the electrode by electrolyte ions^41–43^.

### First-principle study

#### The effect of functionalization on quantum capacitance of GO

The total interfacial capacitance of graphene-based supercapacitors is given by Eq. (5):

5 $$\frac{1}{{C}_{t}}=\frac{1}{{C}_{D}}+\frac{1}{{C}_{Q}}$$

where C_t_, C_D_, and C_Q_, are the total capacitance, the EDLC capacitance, and the quantum capacitance, respectively.

For a 2D material, the integrated quantum capacitance can be obtained by Eq. (6):

6 $$ C_{Q} = e^{2} \mathop \smallint \limits_{ - \infty }^{ + \infty } D_{E} \left( T \right)F_{T} \left( {E - e\Phi_{g} } \right)dE $$

where *e*, *D*(*E*), *F*_*T*_(*E*)*,* and Φ represent elementary electric charge, the density of states, the thermal broadening function, and the potential, respectively. Also, *F*_*T*_(*E*) is given by Eq. (7):

7 $$ F = \left( {4k_{{\text{B}}} T} \right) {\text{sech}}^{2} \left( {E/2k_{{\text{B}}} T} \right) $$

where *k*_B_ is the Boltzmann constant and T is the temperature that is usually chosen to be room temperature (300 K)^44,45^.

**Figure 11:**
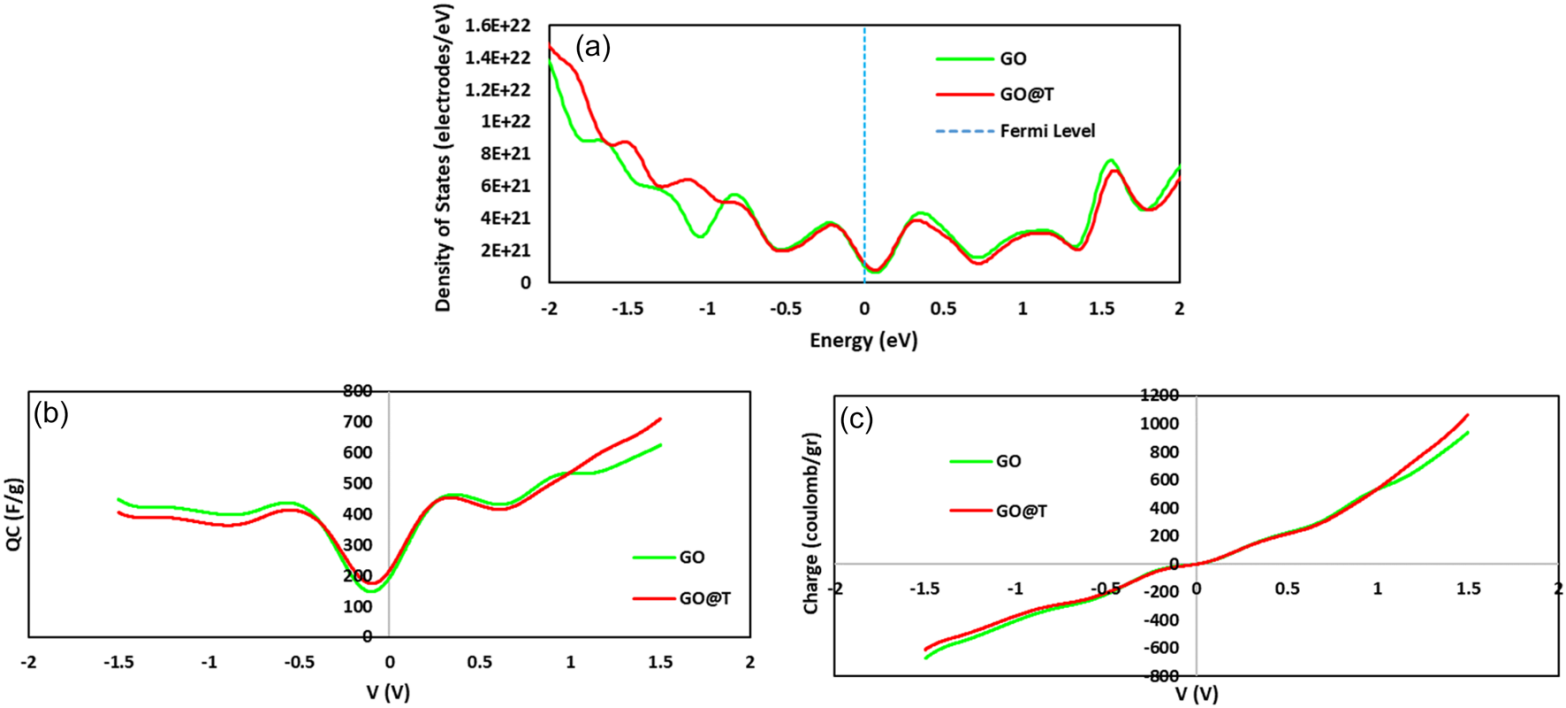
The plots of (**a**) DOS, (**b**) integrated quantum capacitance, and (**c**) net charge.

As can be seen from density of states (DOS), the results of the calculations show that the quantum capacitance increases with tris functionalization. The quantum capacitance of GO@T in the range of water stability voltage (− 0.62–0.83 V) is 368.0, 468.5 Fg^− 1^ compared to 293.3, 383.6 Fg^− 1^ for GO (Fig. 11).

#### Gibbs free energy calculation of functionalization

The Gibbs free energy difference (ΔG) can be calculated using Eq. (8):

8 $$\Delta G={\Delta E}_{tot}+{\Delta E}_{ZPE}-T\Delta S$$

where ΔE_tot_ is the total DFT energy difference, ΔE_ZPE_ is the zero-point energy correction difference, and TΔS is the entropy changes under constant temperature and pressure (T = 298.15 K, P = 1 atm) which can be ignored because the system involves condensed phases^46^. The energy values for each species are shown in Table 1. The results indicated that the Gibbs free energy difference of the amide reaction is equal to 0.03 eV.

**Table 1:** The obtained energy and E_ZPE_ of the compounds.

Sample	E (eV)	E_ZPE_ (eV)
GO	− 122,129.051	18.899
T	− 11,949.295	4.396
GO@T	− 131,999.911	22.643
H_2_O	− 2078.319	0.569

#### The effect of functionalization on GO re-stacking

The distance between the functionalized sheets depends on the size of the functional group. Therefore, it seems that the distance between the layers is affected by substituting the functional groups of graphene oxide by tris.

**Figure 12:**
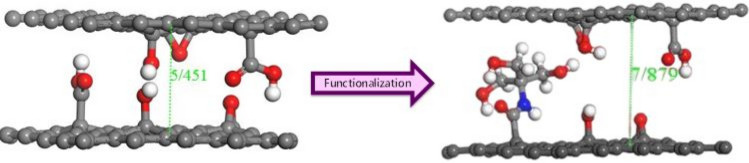
Distance between two graphene oxide layers before and after functionalization with tris.

To investigate the distance between the functionalized sheets, two layers of GO, and two layers of GO@T were optimized. The calculation results clearly show that the distance between graphene oxide layers increased after functionalization. As can be seen in Fig. 12, the distance between the layers before and after functionalization is 5.451 and 7.879 Å, respectively. Increasing the surface area and decreasing the re-stacking will increase the capacity of the double layer because more surface area will be available to the electrolyte materials, as reported in previous works^33^.

## Experimental section

### Materials

Graphite powder (CAS No: 7782-42-5), sodium nitrate (CAS No: 7631-99-4), potassium permanganate (CAS No: 7722-64-7), sulfuric acid (CAS No: 7664-93-9), hydrogen peroxide (CAS No: 7722-84-1), hydrochloric acid (CAS No: 7647-01-0), tris(hydroxylmethyl)aminomethane (CAS No: 77-86-1), dicyclohexylcarbodiimide (DCC) (CAS No: 538-75-0), 4-dimethylaminopyridine (DMAP) (CAS No: 1122-58-3), and acetonitrile (CAS No: 75-05-8) solvent were used for graphene oxide functionalization. Sodium sulfate (CAS No: 7757-82-6) was used as electrolyte. All chemicals utilized in this work were commercially accessible and were utilized without further purification.

### Preparation of graphene oxide

Graphene oxide was synthesized using a modified Hummers method. The graphite powder (1 g) and sodium nitrate (0.5 g) were dissolved in 98% sulfuric acid (20 mL) in weight ratio of 2:1. The suspension was sonicated at 66 °C for 36 min and then stirred at 20 °C for 1 h. Then, potassium permanganate powder (3 g) was slowly added into the suspension with continuous stirring at 81 and 95 °C for 1 h. After that, 50 mL distilled water was carefully added to the mixture and heated at 100 °C for 1 h. The suspension was poured into distilled water (24 mL) and then hydrogen peroxide (12 mL) was added slowly. To ensure the removal of reaction side products, the product was washed with 37% hydrochloric acid and distilled water. The obtained product was dried in the oven at 70* °*C for 24 h.

## Preparation of graphene oxide functionalized with tris(hydroxymethyl)aminomethane

**Figure 13:**
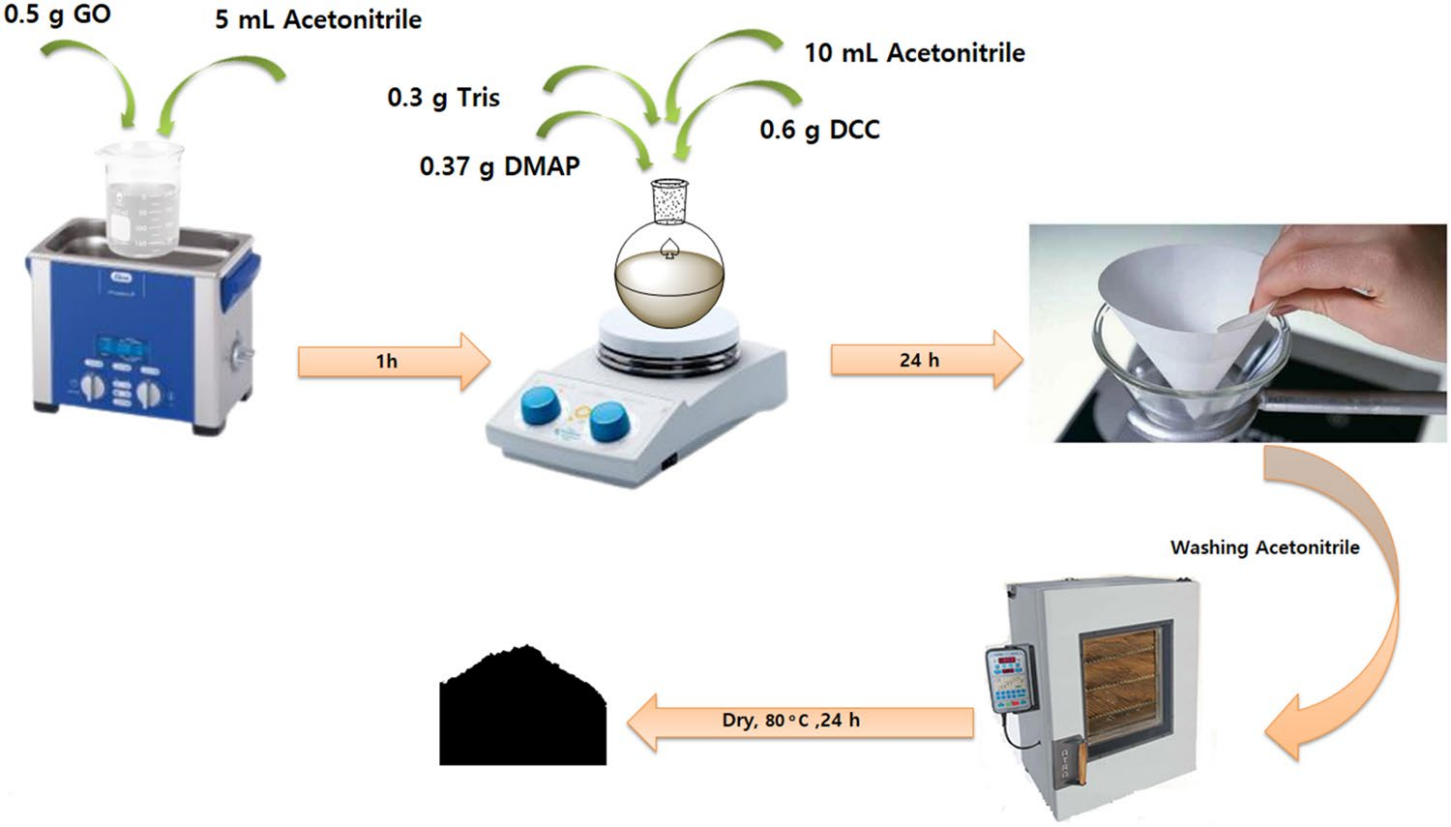
General procedure for the preparation of GO@T.

Initially, tris (0.3 g), DCC (0.6 g), and DMAP (0.37 g) were dissolved in 10 mL acetonitrile followed by stirring for 1 h at room temperature. In the next step, the GO (0.5 g) was dispersed in 5 mL acetonitrile solvent and then added to the previous solution and stirred at room temperature for 24 h. The obtained precipitate was washed several times with acetonitrile and finally dried in an oven at 80 °C (Fig. 13).

### Electrochemical measurements

In order to investigate the electrochemical behavior, galvanostatic Autolab device model PGSTAT204 was used for cyclic voltammetry and impedance spectroscopy. KIMIA STAT device model 126 was also used for charge/discharge tests. In the three-electrode system for electrochemical tests, platinum foil, standard Ag/AgCl electrode, and graphite plate coated with the active material were used as the counter electrode, reference electrode, and working electrode, respectively. Sodium sulfate solution (0.5 M) was used as the electrolyte. In order to prepare the working electrode, the active material, carbon black, and polytetrafluoroethylene with the ratio of 80:15:5 were first mixed and then dispersed in ethanol solution. The suspension was homogenized for 30 min in ultrasonic bath. This homogenized slurry was coated uniformly on a graphite electrode by painting method and dried for 12 h at 70 °C. About 0.15–0.50 mg of the electrode material was loaded on the graphite substrate. Electrochemical impedance spectroscopy (EIS) was carried out at open circuit potential over the frequency range of 100 kHz to 10 mHz. In order to obtain the electrode capacitance, a galvanostatic charge/discharge method was used in an obtained potential window (− 0.5–0.5 V vs. Ag/AgCl) from cyclic voltammetry.

### Computational section

#### First-principle calculation

All the calculations were performed by utilizing DFT within the plan-wave pseudopotential plot accessible in Quantum-ESPRESSO code computer program. To study the distance between two layers of GO and GO@T, the structures were first optimized via the generalized gradient approximation (GGA) in the scheme of Perdew–Burke–Ernzerhof (PBE) exchange–correlation functional^44^. Calculations were performed by making use of a supercell containing a 3 × 3 GO sheet (114 atoms) and a tris molecule (19 atoms). All the supercells were built with a vacuum space of 20 Å along the z-direction to avoid periodic interaction. The 1 × 1 × 1 and 10 × 10 × 1 Monkhorst–Pack k-point sampling for the Brillouin zone is applied for geometry optimizations and DOS calculations respectively.

## Conclusion

The graphene oxide functionalized with tris(hydroxymethyl)aminomethane was prepared as a new material for supercapacitors. The spectroscopic analysis confirmed the successful formation of the GO@T structure. The cyclic voltammetry of the samples showed that the redox reaction does not occur. GCD tests showed a specific capacitance of 549.8 F g^− 1^ at a specific current of 2.5 A g^− 1^ in a 0.5 M Na_2_SO_4_ electrolyte at potential range of − 500–500 mV versus Ag/AgCl. This capacitance, which arises from the electrical double‐layer capacitance, may be the fundamental reason for the improved electrochemical behavior due to the best dispersion of tris-functionalized graphene oxide sheets in comparison to GO. Also, the prepared GO@T showed good cyclic stability, so that after 9300 continuous charge/discharge cycles at the specific current of 7 A g^−1^, about 68% of its initial capacitance was retained.

DFT calculations showed that the distance between the layers has increased from 5.451 Å in the GO to 7.879 Å in GO@T. The quantum capacitance of GO@T in the range of water stability voltage (− 0.62–0.83 V) was also increased.

### Supplementary Information

Supplementary Information.

## Data Availability

All data generated or analyzed during this study are included in this published article [and its supplementary information files].
