# Supplementary material for: An experimental and computational study of graphene oxide functionalized with tris(hydroxymethyl)aminomethane as an electrode material for supercapacitors
Source: Sci Rep. 2023 Oct 5;13:16756. doi: 10.1038/s41598-023-44048-z (PMC10556013; doi:10.1038/s41598-023-44048-z)
Supplement: Supplementary file 1 — Supplementary Information. [file 41598_2023_44048_MOESM1_ESM.docx]

**An experimental and computational study of graphene oxide functionalized with tris(hydroxymethyl)aminomethane as an electrode material for supercapacitors**

Samira Mohammadi, S. Morteza Mousavi‑Khoshdel ^*,1^

*^1^ Industrial Electrochemical Research Laboratory, Department of Chemistry, Iran University of Science* *and Technology, P.O. Box: 16846-13114, Tehran, Iran*

*^*^Email: mmousavi@iust.ac.ir*

**Fig. S1.** The Amide reaction mechanism of GO@T.


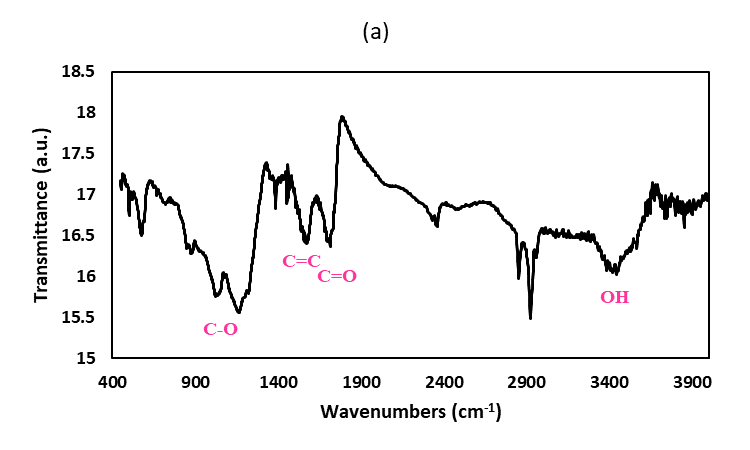

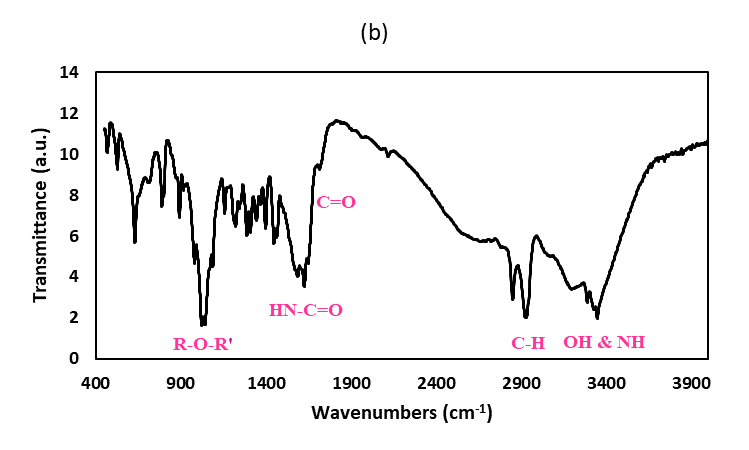


**Fig. S2.** FTIR spectra of (**a**) GO (**b**) GO@T.


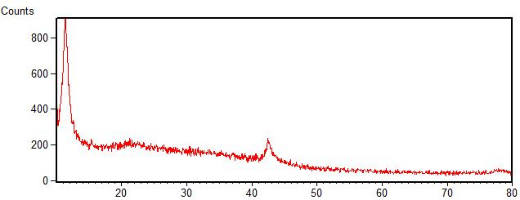


(a)


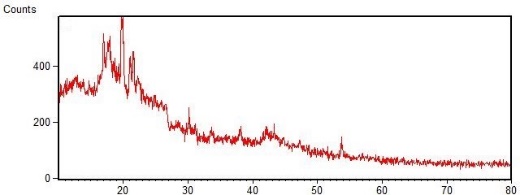


(b)

2θ

2θ

**Fig. S3.** XRD pattern of (a) GO and (b) GO@T.

**Fig. S4.** EDX spectra of GO@T**.**


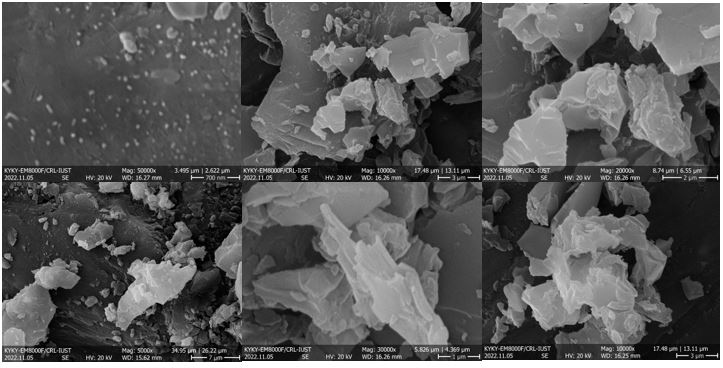


**Fig. S5.** FESEM images of GO@T.


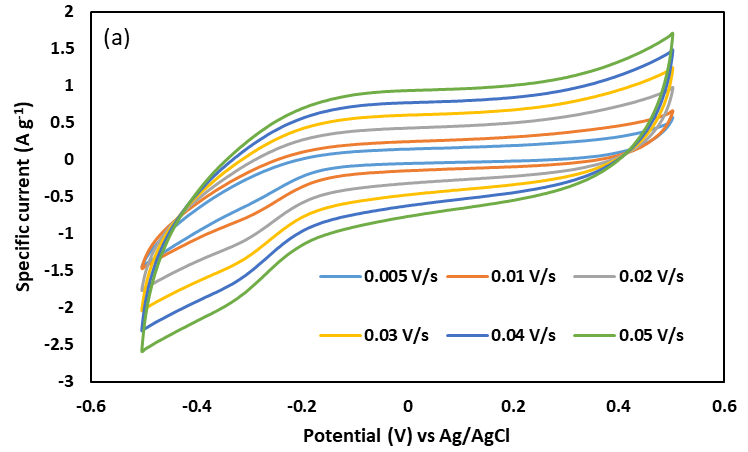


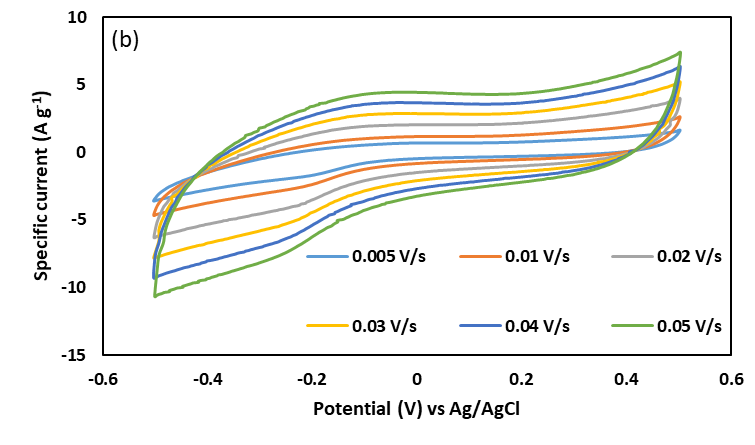

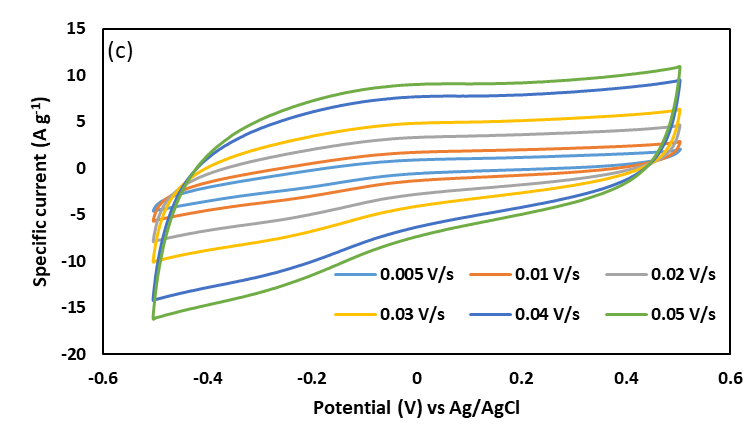


**Fig. S6.** CV curves obtained at different scan rates for: (**a**) tris (**b**) GO (**c**) GO@T.


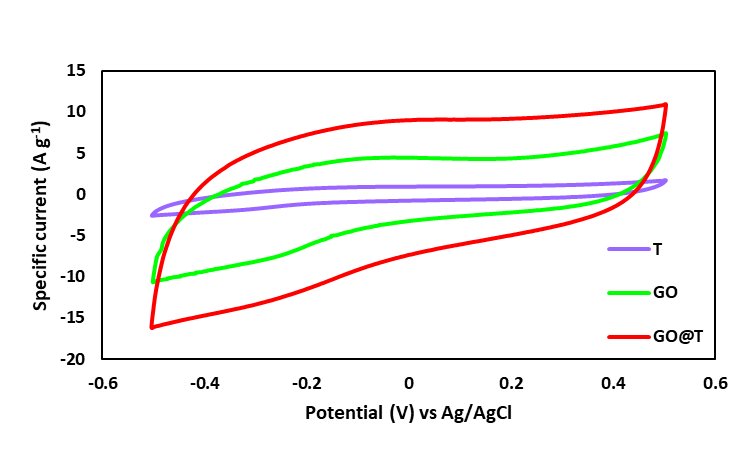


**Fig. S7.** CV curves of T, GO and GO@T at the scan rate of 0.05 *V s^-1^*.


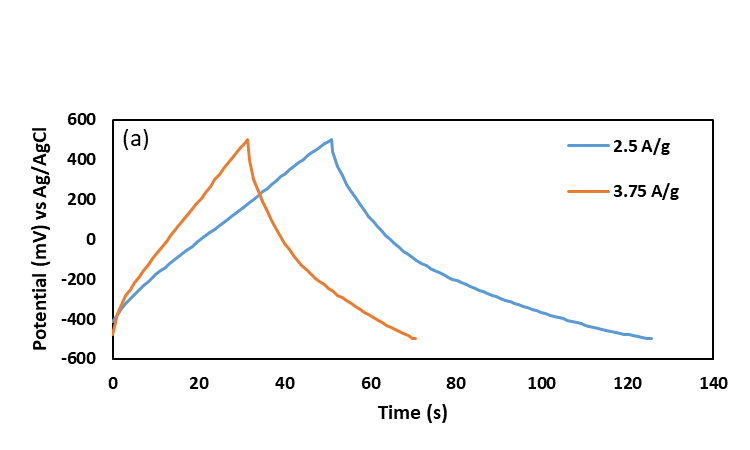


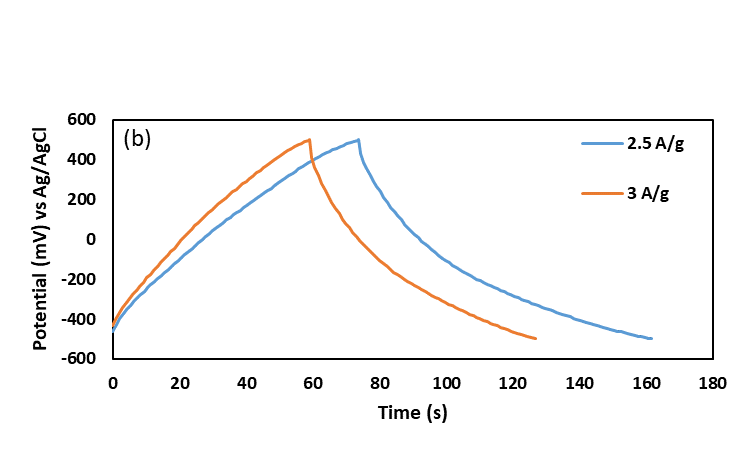

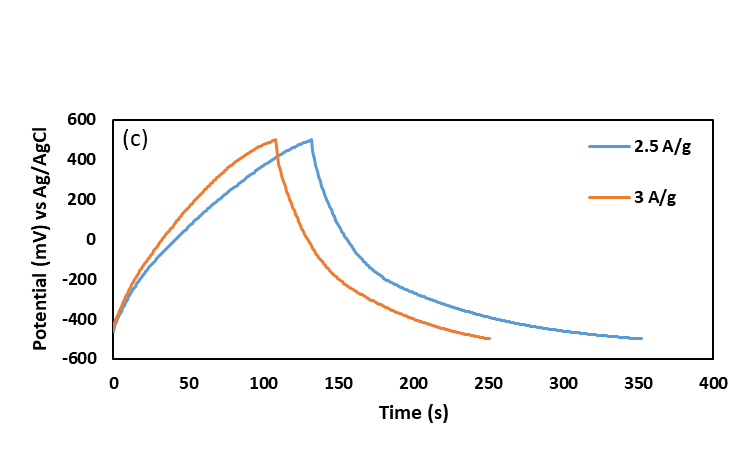


**Fig. S8.** Galvanostatic charge/discharge diagram of (**a**) tris (**b**) GO (**c**) GO@T at low specific currents.


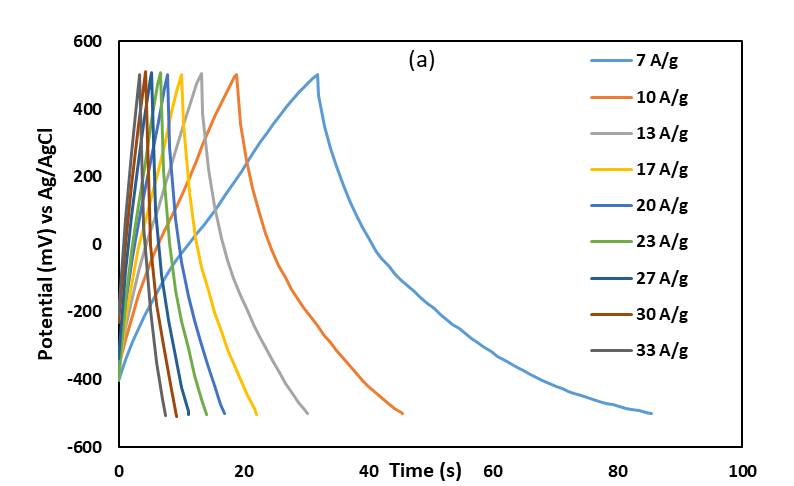


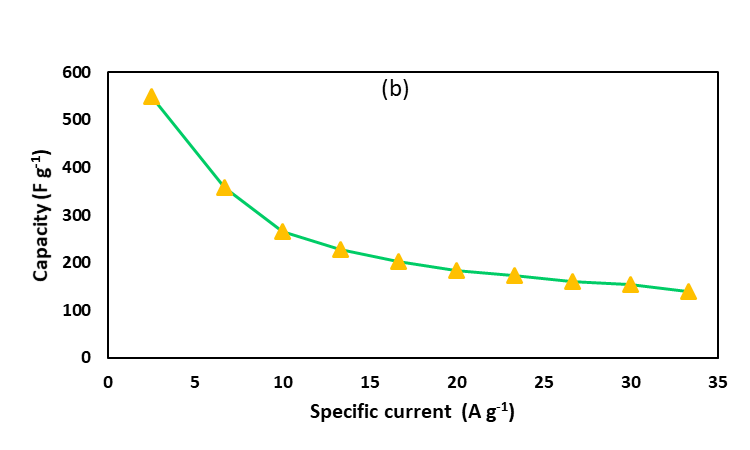

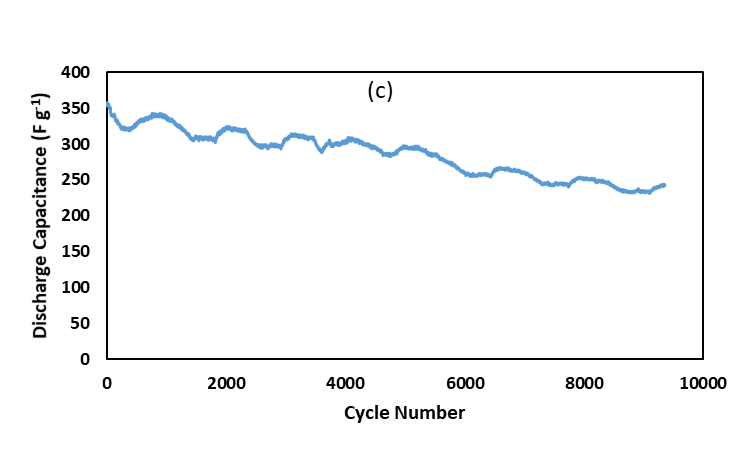


**Fig. S9.**  (**a**) Galvanostatic charge/discharge diagram of GO@T at high specific current, (**b**) specific capacity vs. various specific currents, and (**c**) cyclic stability of GO@T electrode at the specific current of 7*A g^-1^*.


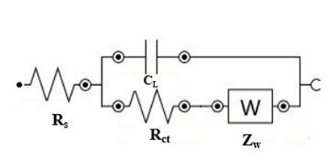

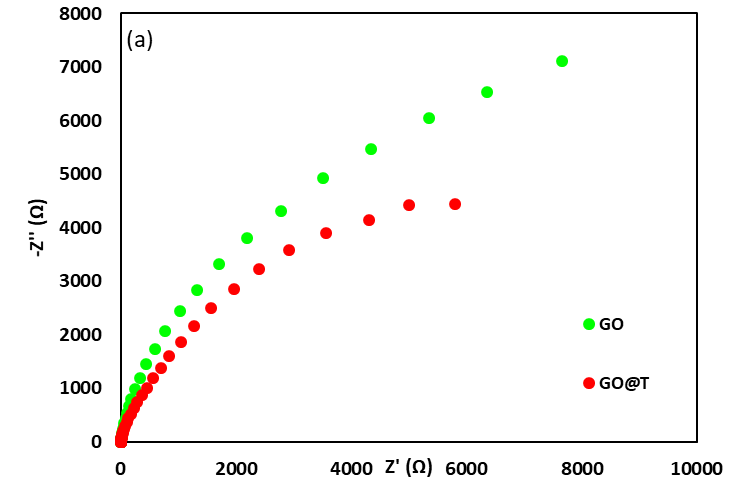

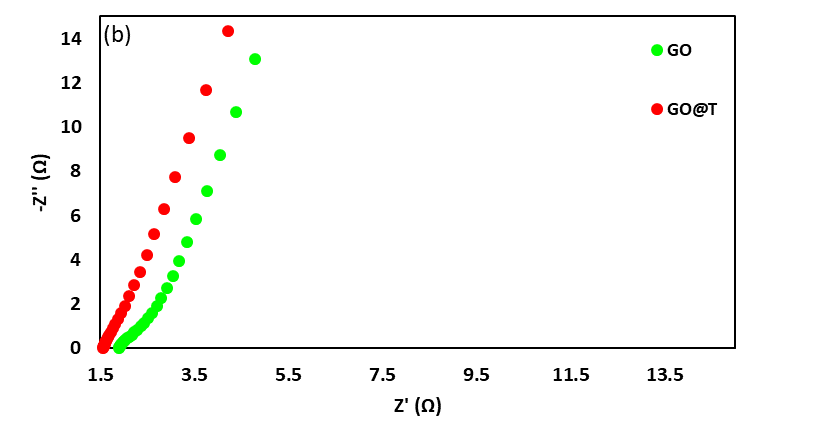


**Fig. S10.** (**a**) Nyquist plots, (**b**) higher magnification of the Nyquist plots at high frequencies

and the electrical equivalent circuit.


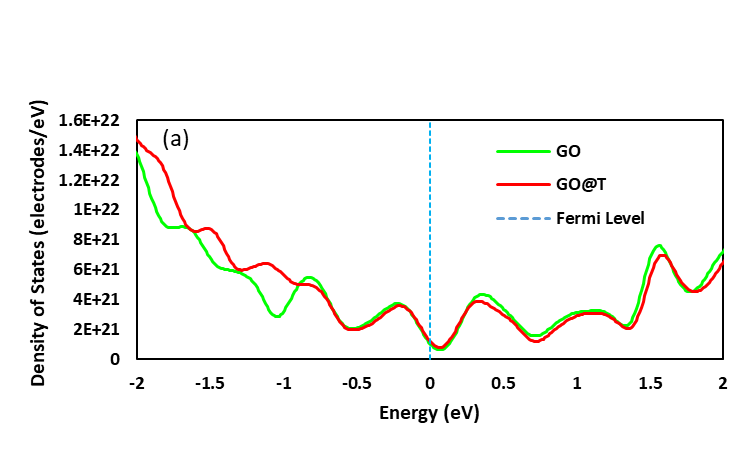


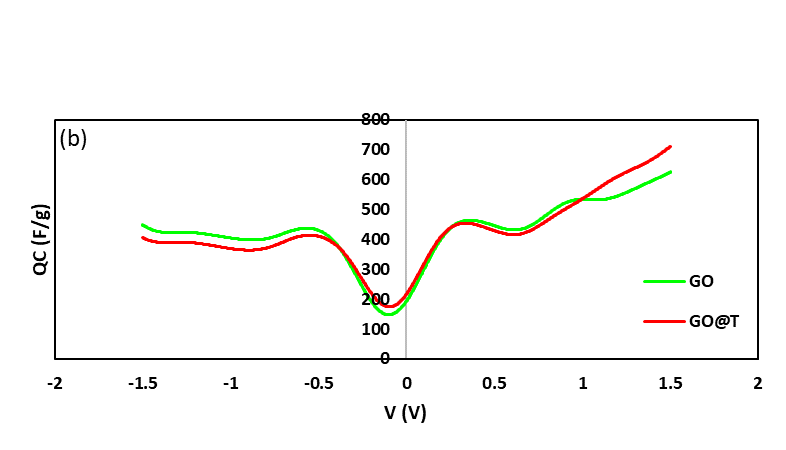

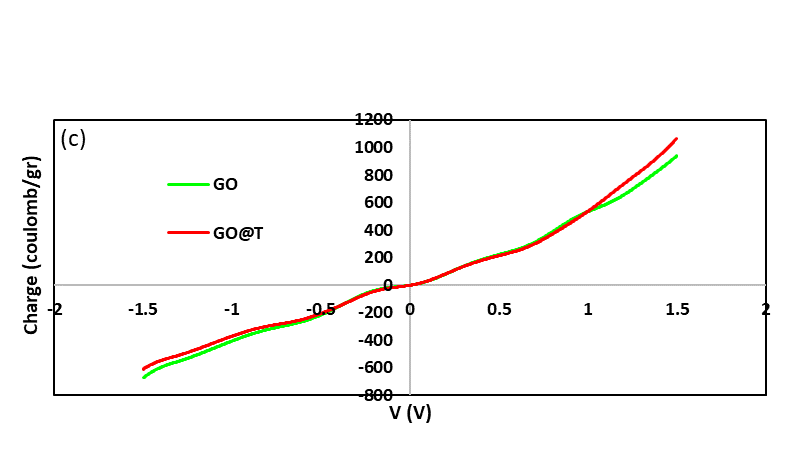


**Fig. S11.** The plots of (**a**) DOS, (**b**) integrated quantum capacitance and (**c**) net charge.


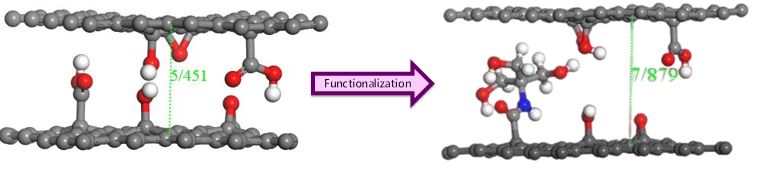


**Fig. S12.** Distance between two graphene oxide layers before and after functionalization with tris.


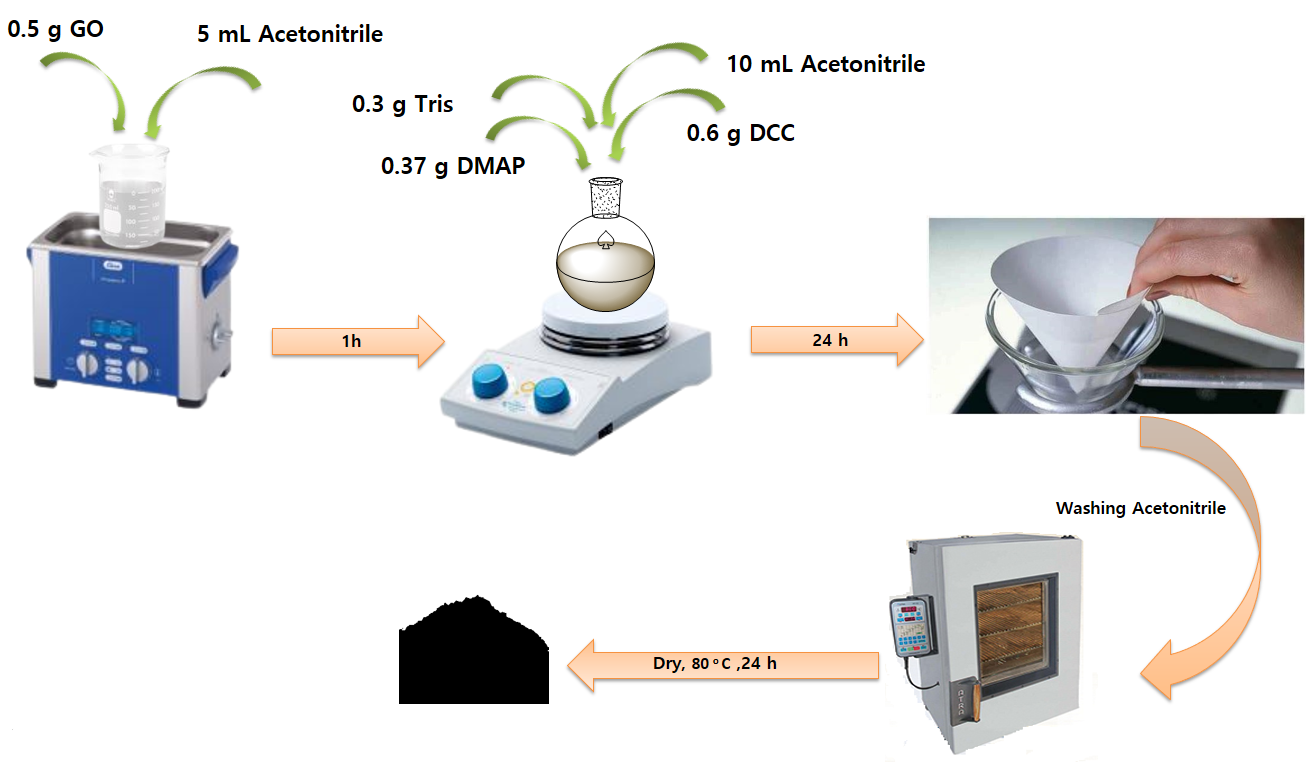


**Fig. S13.** General procedure for the preparation of GO@
